# Effects of normalization on quantitative traits in association test

**DOI:** 10.1186/1471-2105-10-415

**Published:** 2009-12-14

**Authors:** Liang Goh, Von Bing Yap

**Affiliations:** 1 Cancer & Stem Cell Biology Program, Duke-National University of Singapore Graduate Medical School, Singapore; 2 Department of Statistics and Applied Probability, National University of Singapore, Singapore

## Abstract

**Background:**

Quantitative trait loci analysis assumes that the trait is normally distributed. In reality, this is often not observed and one strategy is to transform the trait. However, it is not clear how much normality is required and which transformation works best in association studies.

**Results:**

We performed simulations on four types of common quantitative traits to evaluate the effects of normalization using the logarithm, Box-Cox, and rank-based transformations. The impact of sample size and genetic effects on normalization is also investigated. Our results show that rank-based transformation gives generally the best and consistent performance in identifying the causal polymorphism and ranking it highly in association tests, with a slight increase in false positive rate.

**Conclusion:**

For small sample size or genetic effects, the improvement in sensitivity for rank transformation outweighs the slight increase in false positive rate. However, for large sample size and genetic effects, normalization may not be necessary since the increase in sensitivity is relatively modest.

## Background

Genome-wide association (GWA) studies have been used to identify over 200 potential causal loci in complex diseases such as metabolic/cardiovascular disorder, autoimmune disorder, and cancer [1-6]. The approach requires a stringent adherence to quality control, statistical analyses and replication studies [7]. In quantitative trait loci (QTL) analysis, there is an implicit assumption that the phenotype data follow a normal distribution. Violation of this assumption can severely affect the power and type I error [8].

In most cases, quantitative traits are not normally distributed and one strategy is to perform parametric transformation to approximate normality. In cases where the traits are left-skewed, the appropriate transformation may not be so straight-forward. Transformation is crucial for meta-analyses, where two or more populations are combined to improve statistical power. Preferably, the traits are transformed similarly to enable comparison of genetic effects. This may prove challenging when the traits from different populations are not similarly distributed and cannot be transformed in the same manner. Literature search in this area shows evaluation of normalization methods in pedigree studies [9-11], looking at the logarithm, power, and rank-based transformations, and giving mixed results. In Labbe and Wormald [10], Box-Cox and log were compared and Box-Cox was found to perform better. Peng et al [11] evaluated ENQT, a rank-based transformation in variance components and semi-parametric QTL models, showing ENQT improved power. For Diao and Lin [9], the question was not which normalization worked best but whether the semi-parametric QTL performed better than the variance-components models in terms of power and type I error, as well as robustness to outliers when the assumption of normality does not hold. Nevertheless, Diao and Lin's simulations showed that estimated and true transformations (i.e. which restored normality) gave higher power and lower Type I error for likelihood-ratio tests compared to the logarithm, the square-root (a special case of Box-Cox) and untransformed data.

In the absence of comprehensive study on various transformation methods for association studies, it is difficult to decide which transformation to apply. How much normality will suffice for the underlying assumption of association tests? In the context of sample size and genetic effects, what are the effects of these methods? Although it is generally accepted that deviation from normality can reduce the power of the study, the effects of different normalization methods are not clearly understood.

In this paper, we report the effects of these normalization methods in basic quantitative association tests on simulated data. Our objective was to quantify the effects of normalization in GWA studies. Since a large sample is needed to detect small effects, we also evaluated the effects of normalization on varying sample sizes and genetics effects. For simplicity, we limit our study on additive models. We also approached the evaluation by emulating GWA studies, measuring performance of the normalization based on the sensitivity of discovering causal single nucleotide polymorphism (SNP) and its respective ranking in association tests after Bonferroni corrections.

## Results

**Figure 1:**
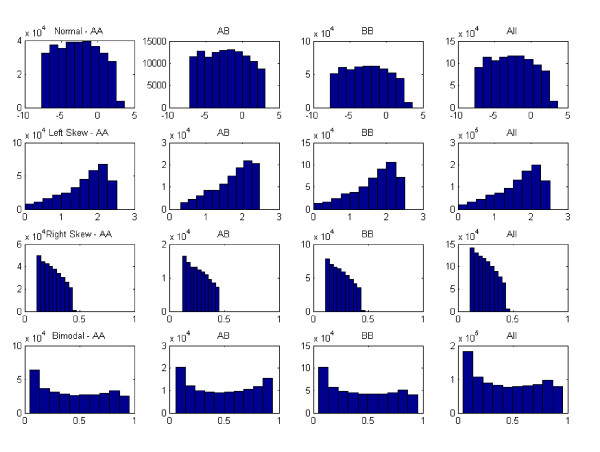
**Quantitative traits generated from HAPSIMU for 8000 samples and PV = 0.01**. From top row: normal, left-skew, right-skew, and bimodal. First 3 columns show the distribution for each genotype (AA, AB, BB) and the last column show the overall distribution. Left-skew was generated using logarithm transformation, and right-skew and bimodal with beta transformation.

**Figure 2:**
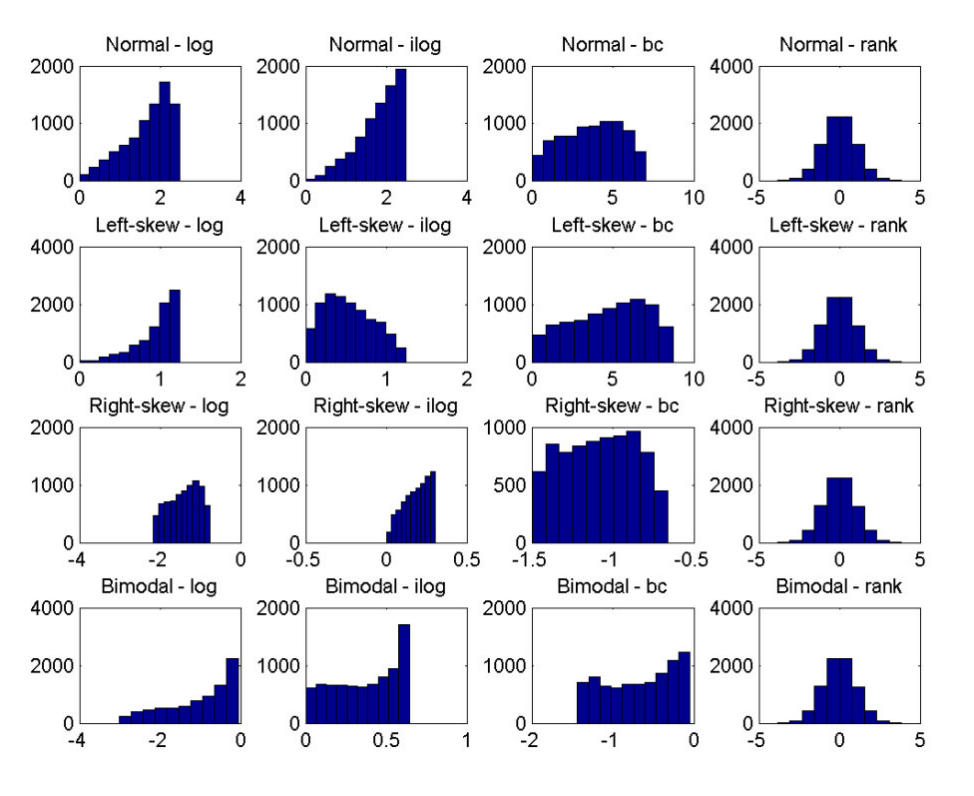
**Transformation distributions of an example data from the dataset of quantitative traits for 8000 samples and PV = 0.01**. From top to bottom: normal, left-skew, right-skew, and bimodal. From left to right: after logarithm (log), inverse-logarithm (ilog), Box-Cox (bc), and rank transformation.

**Figure 3:**
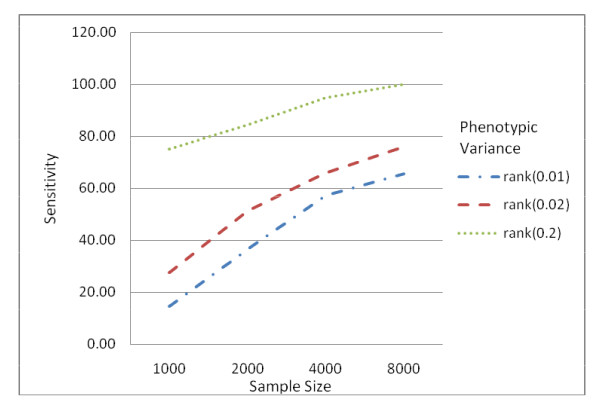
**Effects of PV and sample size for rank-based transformation**. The larger the genetic effects and sample size, the higher the sensitivity.

To assess the effects of normalization, genotype data was generated from a disease model with one causal SNP allele using HAPSIMU [12]. For each sample size and genetic effect, 120 datasets were generated. The average minor allele frequency (MAF) for the causal SNP was 0.077. Four traits, 4 transformations, 4 samples sizes and 3 genetic effects were evaluated. Figure 1 shows the distribution of genotype-specific traits in the simulated dataset for sample size of 8000 and 0.01 phenotypic variance explained (PV). Figure 2 shows the traits distributions of an example data from this dataset after each transformation. Figure 3 shows the sensitivity of rank-based transformation for each sample size and PV. Table 1 shows the sensitivity, false positive rate, and displacement of causal SNP for PV of 0.01. The same trend was observed for other PV of 0.02 and 0.2 (see Additional file 1). In GWA studies, besides genome-wide significance p-value after multiple testing corrections, the ranking of the causal SNP is also important. Preferably, the causal SNP should be ranked high in the list. The displacement is the rank of the causal SNP, minus 1, the expected rank.

**Table 1:** Performance of simulations with PV = 0.01 (small effects) for 4 sample sizes and 4 quantitative traits (normal, left-skew, right-skew, bimodal) transformed using logarithm (log), inverse-logarithm (ilog), Box-Cox, and rank-based.

	True Positive Rate				False Positive Rate				Displacement			
	
	1000	2000	4000	8000	1000	2000	4000	8000	1000	2000	4000	8000
**Normal**	4.27	18.26	43.59	50.86	0.03	0.11	0.10	0.21	24.03	17.11	15.06	10.04
**Left-skew**	5.98	18.26	47.86	53.45	0.09	0.07	0.16	0.17	23.45	15.25	14.24	9.01
**Right-skew**	3.42	20.00	41.88	51.72	0.04	0.11	0.10	0.22	24.16	17.57	15.15	10.28
**Bimodal**	2.56	10.43	35.04	44.83	0.03	0.09	0.09	0.17	26.32	19.16	16.14	11.31

**Normal - Log**	5.98	18.26	47.86	53.45	0.09	0.07	0.16	0.17	23.45	15.25	14.24	9.01
**Normal - ilog**	7.69	27.83	50.43	57.76	0.17	0.21	0.18	0.37	21.91	15.87	13.51	9.55
**Normal - boxcox**	5.13	18.26	44.44	50.86	0.04	0.10	0.12	0.20	23.73	16.87	14.81	10.02
**Normal - rank**	14.53	36.52	57.27	65.52	0.08	0.17	0.24	0.44	18.97	12.50	11.65	6.65

**Left-skew - log**	7.69	22.61	50.43	56.03	0.10	0.07	0.22	0.20	23.08	14.50	13.44	8.16
**Left-skew - ilog**	5.13	19.13	45.3	54.31	0.05	0.09	0.15	0.19	23.46	16.08	14.43	9.58
**Left-skew - boxcox**	5.13	18.26	43.59	50.00	0.05	0.08	0.11	0.17	23.97	16.87	14.71	10.06
**Left-skew - rank**	14.53	36.52	57.27	65.52	0.08	0.17	0.24	0.44	18.97	12.50	11.65	6.65

**Right-skew - log**	5.13	16.52	41.88	49.14	0.04	0.08	0.12	0.17	23.99	17.09	14.83	10.21
**Right-skew - ilog**	3.42	22.61	43.59	51.72	0.05	0.11	0.10	0.23	23.98	17.63	15.17	10.41
**Right-skew - boxcox**	4.27	16.52	41.88	50.86	0.03	0.09	0.10	0.21	24.2	17.23	15.11	10.13
**Right-skew - rank**	14.53	36.52	57.27	65.52	0.08	0.17	0.24	0.44	18.97	12.50	11.65	6.65

**Bimodal - log**	4.27	13.91	41.88	47.41	0.08	0.05	0.13	0.15	25.32	16.84	14.91	10.35
**Bimodal - ilog**	2.56	11.30	35.90	45.69	0.04	0.10	0.08	0.19	26.25	19.35	16.26	11.35
**Bimodal - boxcox**	2.56	12.17	36.75	44.83	0.04	0.08	0.08	0.17	25.71	18.07	15.48	10.8
**Bimodal - rank**	14.53	36.52	57.27	65.52	0.08	0.17	0.24	0.44	18.97	12.50	11.65	6.65

## Discussion

A GWA study is an expensive venture which requires careful design and quality control. One such consideration is normalization. Our simulation indicated that in studies where the effects and sample size were large; transformation had little effect. Performance issues arise when the genetic effect was less than moderate and sample size was not large. In this case, the type of normalization became a serious consideration with impacts on results.

The results showed that larger PV (more genetic effects) and samples size generally gave higher sensitivity and smaller displacement with a slight increase in false positive rate. When PV varied from 0.01 to 0.2, untransformed normal distribution showed sensitivity of 4.27% to 50.86% (PV = 0.01), 13.27% to 64.96% (PV = 0.02), and 71.79% to 97.44% (PV = 0.2) for 1000 to 8000 samples (see Table 1 and Additional file 1). When the phenotype was rank transformed, the sensitivity ranged from 14.53% to 65.52% (PV = 0.01), 27.43% to 76.07% (PV = 0.02) and 75.21% to 100% (PV = 0.2) respectively. This is in concordance with literature; with larger sample size and genetic effects, the causal SNP is more likely to be discovered. It also showed improved results with rank-based transformation for all phenotypes, though the amount of improvement depends on sample size and PV. The improvement factor for sensitivity (i.e. sensitivity_rank-based_/sensitivity_untransformed_) varied from 2.43 to 5.67 (1000 samples), 1.83 to 3.50 (2000 samples), 1.2 to 1.63 (4000 samples), 1.23 to 1.46 (8000 samples) for PV = 0.01. For PV = 0.2, the improvement factor ranged from 1.04 to 1.11 (1000 samples), 1.02 to 1.11 (2000 samples), 1.07 to 1.17 (4000 samples), and 1.03 to 1.06 (8000 samples). The improvement factor tended towards 1 for PV = 0.2, indicating that for larger genetic effects, transformation was not so critical (the sensitivity improved marginally but with a higher false positive). This trend was also observed in the case of larger sample size regardless of the distribution of original traits.

Displacement of causal SNP decreased with increasing sample size and PV. For PV = 0.01 and 1000 samples of the untransformed trait, the causal SNP was ranked on average 24.03 out of 100. This improved to 18.81 (PV = 0.02) and 5.03 (PV = 0.2). With rank-based transformation, the displacement improved: 18.97 (PV = 0.01), 16.38 (PV = 0.02), and 4.78 (PV = 0.2).

False positive rate is a concern in GWA studies. In our study, the false positive rate increased with PV and sample size. The simulation showed that rank-based transformation incurred a slightly higher false positive compared with the other methods. We recognize that the false positive rate computed here was subjective, depending on the 99 non-causal SNPs in simulated data. Nevertheless, it gave an indication of false positives amongst the different settings in simulations; i.e. traits distribution, sample size, and genetic effects. Does this mean that rank transformation has no benefits? For small sample size and genetic effects, the increase of sensitivity outweighs the slight increase in false positive rate. For large sample size and genetic effects, this is not true so caution is needed on transformation. We propose no transformation in this scenario.

**Figure 4:**
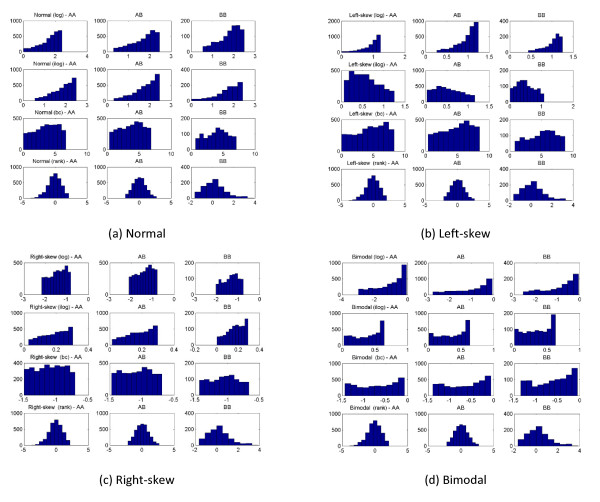
**Genotype distribution of the 4 quantitative traits for 8000 samples and PV = 0.01**. (a) normal, (b) left-skew, (c) right-skew, (d) bimodal after each transformation of logarithm (log), inverse-logarithm (ilog), Box-Cox (bc), and rank-based. Rank-based transformation adjusts the distribution of each genotype to within its tertile while maintaining consistent variance of 1.

Despite the concerns of rank-based transformation being too 'perfect', results showed that it improved the sensitivity and ranking, both important in deciding genes or loci of interest for fine-mapping. One of the reasons sensitivity improved was due to the effects transformation had on the distribution of each genotype. The transformation attempted to keep the distribution of each genotype within its respective tertile (3 genotypes) while maintaining a variance of 1 (Figure 4). In the additive model, the distribution of AA after rank-based transformation veered towards the 33^th ^percentile of the distribution and the BB's distribution towards 67^th ^percentile. The consistent variance of 1 in each genotype meant less overlap between the distributions, accentuating the discriminating additive signals in association test. This also explained why false positive increased correspondingly since the same effects would be applicable on non-causal SNPs.

It was noted in Figure 1 that the normally distributed trait did not follow a perfect normal distribution. In transformation such as log or beta, as long as the original trait followed a normal-like distribution, each trait would transform accordingly to left-skew, right-skew, or bimodal as intended. As for the effects of normality of the original trait on results, we observed that generally the original normal distribution did not perform as well as the rank transformation, suggesting that a 'perfect' normal distribution was desirable at least for small sample size and genetic effects.

In the simulations, Box-Cox sometimes achieved a normal distribution (Figure 2) though not perfectly normal like the rank-based transformation. However, its performance was still not comparable with the latter. In addition, it was observed that although some distribution appeared normal, rank-based transformation could still improve sensitivity. This is interesting as it suggests that it is not just sufficient to attain some form of normality for quantitative traits but to achieve a normality that increases the additive discriminating signals. Transformation should also be considered in the context of sample size and genetic effects. In large sample size and more than moderate effects, it may not be necessary to transform, thus maintaining a lower false positive with comparable sensitivity and displacement. For conditions other than this, rank-based transformation tends to improve the performance regardless of the distribution.

In our literature review, the traits were usually generated as non-normally distributed trait and not well defined traits such as those in our study (left-skew, right-skew, bimodal) where we could assess the effects of transformation on different distributions. This arises from the difficulty of generating quantitative traits to meet these distributions using genotype, especially using real genotype data. In Peng et al (2007), the normal trait was transformed to meet certain skewness and kurtosis for their evaluation. We adopted the same idea in our simulation by transforming the trait after genotype has been generated. This approach has the advantage of allowing us to use existing genotype data (simulated or actual) to study effects of different trait distributions.

## Conclusions

The four quantitative traits investigated here are common distributions described by several statistical empirical distributions. Simulations were done on common factors affecting results of GWA study, such as normalization, sample size, and genetic effects. The simulation showed that rank-based transformation gave the best performance in terms of sensitivity and displacement regardless of the distribution of the trait. This is however accompanied by a slight increase in false positive rate. The positive effect of rank-based transformation decreased with increasing sample size and genetic effects. For large sample size and genetic effects, normalization is not recommended.

## Methods

The normalization methods investigated were: logarithm, Box-Cox, inverse-logarithm (i.e. *log *[-[*x*_*i*_-*min(x)]] *where *x*_*i *_is the quantitative trait for sample *i*), and rank-based transformation [11]. Ranking of SNPs and its p-value from associative tests after multiple test corrections were used for performance assessment. Motivation for the inverse-logarithm transformation came from one of our quantitative traits where the data contained negative values and was highly skewed to the left, where neither logarithm nor Box-Cox would normalize it appropriately. All the transformations were provided or coded in Matlab.

**Figure 5:**
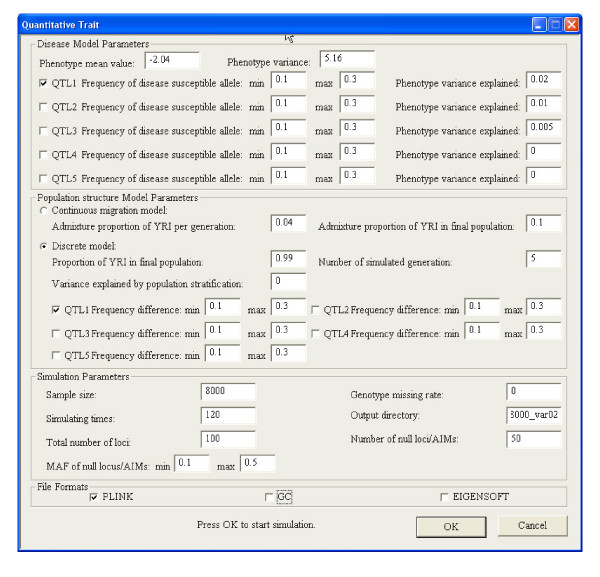
**Parameters setup for HAPSIMU simulation**. The mean and variance in disease model were based on one of the quantitative traits (spherical equivalent) in a myopia project. Population homogeneity was maintained by setting a high proportion of YRI in final population.

HAPSIMU simulates from a model where the effects of the allele are additive based on the phenotypic variance explained (PV) and frequency (f) of the disease susceptibility allele. It utilizes informative marker loci from the ENCODE regions genotyped in CEPH and YRI. A normally distributed trait is generated for each of the genotypes (AA, AB, BB). To avoid confounding factors such as polygenic effects and population admixture, one causal SNP was generated out of 100 SNPs in a homogenous population of YRI for our simulations. We noted that 100 SNPs is not representative of GWAS data, neither is one causal SNP the realistic scenario. However, given that most complex diseases are polygenic model involving probably thousands of causal SNPs, the scenario of 1 causal out of 100 SNPs can be reasonably extrapolated to a GWAS dataset of few thousands causal SNPs; a reasonable representation of a polygenic, additive disease model. The settings for HAPSIMU are shown in Figure 5. 120 sets of data were generated to evaluate the sensitivity and ranking of causal SNP. We also investigated the effects of normalization versus sample size and PV of causal SNP, generating datasets for 1000, 2000, 4000, and 8000 subjects, with PV of 0.01, 0.02, and 0.2. The PV can be seen as having small to large effects on the quantitative traits. In total, 1440 datasets comprising of 100 SNPs (1 causal) was generated.

Quantitative traits generated by HAPSIMU are normally distributed with mean and standard deviation determined by PV and f. To investigate various trait distributions such as left- and right- skew as well as bimodal, traits were log and beta transformed. Four traits were obtained after HAPSIMU (i.e. normal, left-skew, right-skew, bimodal), and each of these traits was transformed using logarithm, inverse-logarithm, Box-Cox, and rank-based, i.e. total of 20 traits. Quantitative traits were tested using the common GWAS software Plink [13] which implemented the likelihood ratio test and Wald test. It generated an output file with extension .qassoc that comprised of estimated regression coefficient, standard error, and asymptotic p-value. The p-value was used for assessment.

Criteria for performance assessment were based on (1) displacement ranking of causal SNP out of the 100 SNPs, where expected rank was 1 (i.e. displacement is 0), and (2) Bonferroni corrected p-value for significant association was <5 × 10^-4^. Significant causal SNPs were considered true positives while significant non-causal SNPs were false positives. Sensitivity or True Positive Rate (TPR) was computed for the 120 datasets using TP/(TP+FN)*100 where TP, FN were true positive and false negative respectively from the confusion table tabulated from 120 datasets. False Positive Rate (FPR) or Type I error was computed as FP/(FP+TN)*100 where FP and TN were false positive and true negative from the confusion table. Since there was only one causal SNP, false negative was either 1 or 0 for each simulation, so sensitivity was synonymous with power.

## Authors' contributions

LG and VBY conceived the study and participated in its design and coordination. LG wrote the Matlab code, performed the analyses and drafted the manuscript. All authors read, edited, and approved the final manuscript.

## Supplementary Material

Additional file 1 **Supplementary Tables**. Table S1: Performance of simulations with PV = 0.02 (moderate-small effects) for 4 sample sizes and 4 quantitative traits (normal, left-skew, right-skew, bimodal) transformed using logarithm (log), inverse-logarithm (ilog), Box-Cox, and rank-based. Table S2: Performance of simulations with PV = 0.2 (large effects) for 4 sample sizes and 4 quantitative traits (normal, left-skew, right-skew, bimodal) transformed using logarithm (log), inverse-logarithm (ilog), Box-Cox, and rank-based. Click here for file
