# Supplementary material for: Effects of normalization on quantitative traits in association test
Source: BMC Bioinformatics. 2009 Dec 14;10:415. doi: 10.1186/1471-2105-10-415 (PMC2800123; doi:10.1186/1471-2105-10-415)
Supplement: Additional file 1 — Supplementary Tables. Table S1: Performance of simulations with PV = 0.02 (moderate-small effects) for 4 sample sizes and 4 quantitative traits (normal, left-skew, right-skew, bimodal) transformed using logarithm (log), inverse-logarithm (ilog), Box-Cox, and rank-based. Table S2: Performance of simulations with PV = 0.2 (large effects) for 4 sample sizes and 4 quantitative traits (normal, left-skew, right-skew, bimodal) transformed using logarithm (log), inverse-logarithm (ilog), Box-Cox, and rank-based. [file 1471-2105-10-415-S1.DOC]

Additional file 1

Table S1 Performance of simulations with PV=0.02 (moderate-small effects) for 4 sample sizes and 4 quantitative traits (normal, left-skew, right-skew, bimodal) transformed using logarithm (log), inverse-logarithm (ilog), Box-Cox, and rank-based.

|  | True Positive Rate | | | | False Positive Rate | | | | Displacement | | | |
| --- | --- | --- | --- | --- | --- | --- | --- | --- | --- | --- | --- | --- |
|  | 1000 | 2000 | 4000 | 8000 | 1000 | 2000 | 4000 | 8000 | 1000 | 2000 | 4000 | 8000 |
| Normal | 13.27 | 29.57 | 52.99 | 64.96 | 0.04 | 0.11 | 0.28 | 0.44 | 18.81 | 13.23 | 8.46 | 4.73 |
| Left-skew | 15.93 | 33.91 | 53.85 | 67.52 | 0.04 | 0.15 | 0.28 | 0.49 | 17.97 | 11.56 | 7.90 | 4.12 |
| Right-skew | 13.27 | 31.30 | 52.99 | 65.81 | 0.05 | 0.10 | 0.28 | 0.41 | 18.88 | 13.61 | 8.60 | 4.76 |
| Bimodal | 11.50 | 25.22 | 45.30 | 57.27 | 0.04 | 0.09 | 0.23 | 0.36 | 20.15 | 14.80 | 9.75 | 5.63 |
| Normal - Log | 15.93 | 33.91 | 53.85 | 67.52 | 0.04 | 0.15 | 0.28 | 0.49 | 17.97 | 11.56 | 7.90 | 4.12 |
| Normal - ilog | 19.47 | 40.00 | 58.97 | 70.94 | 0.15 | 0.19 | 0.47 | 0.66 | 18.00 | 13.03 | 8.15 | 4.22 |
| Normal - boxcox | 13.27 | 31.30 | 52.99 | 64.96 | 0.05 | 0.11 | 0.28 | 0.44 | 18.56 | 12.83 | 8.38 | 4.60 |
| Normal - rank | 27.43 | 51.30 | 65.81 | 76.07 | 0.06 | 0.23 | 0.47 | 0.73 | 16.38 | 10.90 | 6.50 | 2.81 |
| Left-skew - log | 18.58 | 40.87 | 54.70 | 69.23 | 0.06 | 0.21 | 0.30 | 0.52 | 17.85 | 10.93 | 6.97 | 3.85 |
| Left-skew - ilog | 13.27 | 33.91 | 54.70 | 65.81 | 0.04 | 0.11 | 0.28 | 0.47 | 18.19 | 12.25 | 8.27 | 4.32 |
| Left-skew - boxcox | 13.27 | 31.30 | 52.14 | 64.96 | 0.04 | 0.10 | 0.28 | 0.45 | 18.49 | 12.67 | 8.52 | 4.64 |
| Left-skew - rank | 27.43 | 51.30 | 65.81 | 76.07 | 0.06 | 0.23 | 0.47 | 0.73 | 16.38 | 10.90 | 6.50 | 2.81 |
| Right-skew - log | 13.27 | 30.43 | 52.14 | 64.96 | 0.04 | 0.10 | 0.27 | 0.43 | 18.55 | 12.83 | 8.57 | 4.74 |
| Right-skew - ilog | 13.27 | 32.17 | 52.99 | 65.81 | 0.05 | 0.10 | 0.30 | 0.46 | 18.75 | 13.47 | 8.62 | 4.66 |
| Right-skew - boxcox | 13.27 | 29.57 | 52.99 | 64.96 | 0.04 | 0.11 | 0.26 | 0.42 | 18.87 | 13.23 | 8.55 | 4.77 |
| Right-skew - rank | 27.43 | 51.30 | 65.81 | 76.07 | 0.06 | 0.23 | 0.47 | 0.73 | 16.38 | 10.90 | 6.50 | 2.81 |
| Bimodal - log | 10.62 | 30.43 | 49.57 | 61.54 | 0.04 | 0.11 | 0.26 | 0.41 | 19.07 | 12.34 | 9.03 | 4.92 |
| Bimodal - ilog | 10.62 | 28.70 | 47.86 | 60.68 | 0.04 | 0.10 | 0.26 | 0.40 | 20.14 | 14.57 | 9.65 | 5.52 |
| Bimodal - boxcox | 10.62 | 26.09 | 47.01 | 60.68 | 0.04 | 0.07 | 0.25 | 0.36 | 19.68 | 13.86 | 9.51 | 5.22 |
| Bimodal - rank | 27.43 | 51.30 | 65.81 | 76.07 | 0.06 | 0.23 | 0.47 | 0.73 | 16.38 | 10.90 | 6.50 | 2.81 |

Table S2 Performance of simulations with PV=0.2 (large effects) for 4 sample sizes and 4 quantitative traits (normal, left-skew, right-skew, bimodal) transformed using logarithm (log), inverse-logarithm (ilog), Box-Cox, and rank-based.

|  | True Postive Rate | | | | False Positive Rate | | | | Displacement | | | |
| --- | --- | --- | --- | --- | --- | --- | --- | --- | --- | --- | --- | --- |
|  | 1000 | 2000 | 4000 | 8000 | 1000 | 2000 | 4000 | 8000 | 1000 | 2000 | 4000 | 8000 |
| Normal | 71.79 | 78.26 | 87.18 | 97.44 | 0.52 | 1.18 | 1.88 | 3.27 | 5.03 | 1.97 | 0.22 | 0.04 |
| Left-skew | 67.52 | 75.65 | 81.20 | 94.02 | 0.28 | 0.84 | 1.31 | 2.37 | 5.79 | 1.98 | 0.30 | 0.05 |
| Right-skew | 72.65 | 82.61 | 88.89 | 97.44 | 0.70 | 1.27 | 2.22 | 3.73 | 4.98 | 2.03 | 0.21 | 0.03 |
| Bimodal | 72.65 | 80.87 | 87.18 | 96.58 | 0.55 | 1.14 | 1.81 | 3.19 | 5.35 | 2.47 | 0.25 | 0.06 |
| Normal - Log | 67.52 | 75.65 | 81.20 | 94.02 | 0.28 | 0.84 | 1.31 | 2.37 | 5.79 | 1.98 | 0.30 | 0.05 |
| Normal - ilog | 80.34 | 86.96 | 96.58 | 100.00 | 1.56 | 1.98 | 3.36 | 5.02 | 5.01 | 2.00 | 0.23 | 0.05 |
| Normal - boxcox | 70.94 | 76.52 | 86.32 | 97.44 | 0.43 | 1.11 | 1.74 | 2.99 | 5.32 | 1.99 | 0.23 | 0.05 |
| Normal - rank | 75.21 | 84.35 | 94.87 | 100.00 | 0.68 | 1.37 | 2.50 | 4.16 | 4.78 | 1.48 | 0.17 | 0.04 |
| Left-skew - log | 66.67 | 73.91 | 74.36 | 89.74 | 0.31 | 0.76 | 1.15 | 2.00 | 6.24 | 2.30 | 0.43 | 0.05 |
| Left-skew - ilog | 70.94 | 76.52 | 85.47 | 97.44 | 0.40 | 0.93 | 1.65 | 2.80 | 5.24 | 1.95 | 0.25 | 0.05 |
| Left-skew - boxcox | 70.94 | 76.52 | 85.47 | 97.44 | 0.40 | 0.97 | 1.65 | 2.83 | 5.44 | 1.97 | 0.27 | 0.05 |
| Left-skew - rank | 75.21 | 84.35 | 94.87 | 100.00 | 0.68 | 1.37 | 2.50 | 4.16 | 4.78 | 1.48 | 0.17 | 0.04 |
| Right-skew - log | 70.94 | 75.65 | 85.47 | 97.44 | 0.39 | 0.92 | 1.61 | 2.83 | 5.47 | 1.98 | 0.27 | 0.05 |
| Right-skew - ilog | 73.50 | 82.61 | 91.45 | 98.29 | 0.83 | 1.35 | 2.47 | 3.99 | 4.98 | 2.07 | 0.19 | 0.03 |
| Right-skew - boxcox | 70.94 | 77.39 | 86.32 | 97.44 | 0.43 | 1.10 | 1.78 | 3.08 | 5.23 | 2.03 | 0.23 | 0.04 |
| Right-skew - rank | 75.21 | 84.35 | 94.87 | 100.00 | 0.68 | 1.37 | 2.50 | 4.16 | 4.78 | 1.48 | 0.17 | 0.04 |
| Bimodal - log | 66.67 | 74.78 | 80.34 | 92.31 | 0.27 | 0.77 | 1.22 | 2.28 | 6.20 | 2.12 | 0.40 | 0.06 |
| Bimodal - ilog | 72.65 | 82.61 | 89.74 | 97.44 | 0.66 | 1.32 | 2.05 | 3.57 | 5.35 | 2.59 | 0.24 | 0.07 |
| Bimodal - boxcox | 67.52 | 75.65 | 82.91 | 94.02 | 0.28 | 0.84 | 1.39 | 2.58 | 5.76 | 2.12 | 0.32 | 0.06 |
| Bimodal - rank | 75.21 | 84.35 | 94.87 | 100.00 | 0.68 | 1.37 | 2.50 | 4.16 | 4.78 | 1.48 | 0.17 | 0.04 |
